# Society Meetings

**Published:** 1911-04

**Authors:** 


### SOCIETY MEETINGS

---

At the regular stated meeting of the Buffalo Academy of Medicine held on Tuesday evening, March 21, 1911, the following-named members were nominated for respective offices for the ensuing year as follows: president, Bernard Bartow and Stephen Y. Howell; secretary, Harry W. Church; treasurer, Lawrence Hendee; trustee for three years, John H. Pryor. The election will be held at the annual meeting June 13, 1911.

---

The Buffalo Academy of Medicine held meetings during February and March as follows:

*Section of Obstetrics and Gynecology.*—Tuesday evening, February 28. Program: The midwife, P. W. Van Peyma; Operating room technic, M. D. Mann.

*Section of Surgery.*—Tuesday evening, March 7. Program: Traumatic arthritis as a late complication of fractures of the upper extremity, Prescott LeBreton; Movable cecum as a frequent cause of symptoms in the right iliac region, George T. Tyler; Vicious union after fracture of the shaft of the femur, operative correction, presentation of case—Stereopticon, E. L. Beebe and Prescott LeBreton.

*Section of Medicine.*—Tuesday evening March 14. Program: Gastrointestinal autointoxication, Allen A. Jones.

*Section of Pathology.*—Tuesday evening March 21. Program: Malignant diseases of the kidneys, Henry Adsit; Malignant disease of the prostate, David Wheeler.

---

The Southern Surgical and Gynecological Association at its twenty-third annual session held at Nashville, Tenn., December 13-15, 1910, elected the following named officers for 1910-1911: president, Rudolph Matas, New Orleans; vice-presidents, Guy Leroy Hunter, Baltimore, and T. Garland Sherrill, Louisville; secretary, William D. Haggard, Nashville, (reelected); treasurer, William S. Goldsmith, Atlanta, Ga. The next annual meeting will be held at Washington, D. C., in December, 1911.

---

The Rochester Academy of Medicine held meetings during March as follows:

*Section of Medicine.*—Wednesday evening, March 1. Program: Blood parasites—illustrated with lantern, Frederick G. Novy, University of Michigan.

*Section of Surgery.*—Wednesday evening, March 8. Program: Spinal Anesthesia, William B. Jones.

*Section of Obstetrics.*—Wednesday evening, March 15. Program: The diet and urine in pregnancy, Charles R. Witherspoon; Intravenous medication, description of a simple apparatus, Joseph Roby.

*Section of Public Health.*—Wednesday evening, March 22. Program: The findings in a series of autopsies on insane patients, W. H. Veeder.

---

The Medical Society of the State of New York will hold its one hundred and fifth annual meeting Tuesday and Wednesday, April 18 and 19, 1911, under the presidency of Dr. Charles Stover, of Amsterdam. Dr. J. W. Grosvenor, of Buffalo, is first vice-president, and Dr. Wisner R. Townsend, of New York, is secretary.

